# Bilateral or unilateral tubo-ovarian abscess: exploring its clinical significance

**DOI:** 10.1186/s12905-023-02826-x

**Published:** 2023-12-19

**Authors:** Yael Yagur, Omer Weitzner, Rebecca Shams, Gili Man-El, Yfat Kadan, Yair Daykan, Zvi Klein, Ron Schonman

**Affiliations:** 1 Department of Obstetrics and Gynecology, Meir Medical Center, Kfar Saba, Israel affiliated with The School of Medicine, Tel Aviv University, Tel Aviv, Israel; 2 Department of Gynecology Oncology, Heamek Medical Center, Afula, Israel affiliated with The Ruth and Bruce Rappaport Faculty of Medicine, The Technion-Israel Institute of Technology, Haifa, Israel

**Keywords:** Pelvic inflammatory disease, PID, Tubo-ovarian abscess, TOA, Laparoscopic salpingo-oophorectomy

## Abstract

**Objectives:**

To assess the characteristics of patients with unilateral and bilateral tubo-ovarian abscess (TOA).

**Methods:**

Women diagnosed with TOA during 2003–2017 were included in this retrospective cohort study. TOA was diagnosed using sonography or computerized tomography and clinical criteria, or by surgical diagnosis. Demographics, sonographic data, clinical treatment, surgical treatment, and post-operative information were retrieved.

**Results:**

The study cohort included 144 women who met the inclusion criteria, of whom 78 (54.2%) had unilateral TOA and 66 (45.8%) had bilateral TOA. Baseline characteristics were not different between the groups. There was a statistical trend that women with fewer events of previous PID were less likely to have with bilateral TOA (75.3% vs. 64.1%, respectively; p = 0.074). Women diagnosed with bilateral TOA were more likely to undergo surgical treratment for bilateral salpingo-oophorectomy compared to unilateral TOA (61.5% vs. 42.3%, respectively; p = 0.04). There was no difference in maximum TOA size between groups.

**Conclusions:**

This study detected a trend toward increased need for surgical treatment in women diagnosed with bilateral TOA. These findings may contribute to determining the optimal medical or surgical treatment, potentially leading to a decrease in the duration of hospitalization, antibiotic exposure, and resistance. However, it is important to acknowledge that the results of the current study are limited, and further research is warranted to validate these potential outcomes.

## Introduction

Pelvic inflammatory disease (PID) is an ascending infection of the upper genital tract (uterus, fallopian tubes, and ovaries) that is caused by sexually transmitted organisms [1]. When left untreated, PID can worsen and lead to the formation of an abscess, known clinically as a tubo-ovarian abscess (TOA) [2, 3]. TOA is a serious complication of PID, as it can spread beyond the borders of the upper genital tract to involve adjacent pelvic organs [4].

Among women undergoing treatment for confirmed PID, 15–35% will receive a diagnosis of TOA [5, 6]. PID with progression to TOA is most common during the reproductive years, when sexual activity is usually at its peak and sexually transmitted infections are spread more widely [7–10].

The clinical presentation of this condition includes lower abdominal pain, purulent vaginal discharge, abnormal uterine bleeding, and elevated body temperature. Pelvic examination often reveals cervical, uterine, and adnexal motion tenderness [11]. Ultrasound scan or computed tomography (CT) is the preferred diagnostic imaging used to visualize and characterize a TOA [12, 13]. Diagnosis is usually based on both clinical and imaging parameters [14, 15].

The first-line treatment for TOA is broad-spectrum antibiotics, which successfully eliminate the infection in 70–87% of cases, depending on several factors, including the size of the abscess, patient’s age, and serum levels of inflammatory markers. Failure to respond to pharmacological treatment requires surgical intervention [4, 16–19]. Definitive information on the preferred procedure for TOA is lacking. The choice of surgical intervention depends on factors such as patient fertility planning and the severity of the disease [19, 20]. Options for surgical intervention include drainage, and most often unilateral or bilateral salpingectomy. Unilateral or bilateral oophorectomy and hysterectomy can be an option in selected cases. A laparoscopic approach is preferred to laparotomy in centers with adequate experience [21]. Untreated PID or TOA can lead to long-term complications, such as infertility, ectopic pregnancies, abdominal adhesions, and chronic pelvic pain [22].

Extensive research has been invested in trying to identify the characteristics associated with TOA that were treated surgically versus those that responded well to antibiotics [23–25]. The findings suggested that bilateral as compared to unilateral abscesses were more likely to require surgical intervention [20]. TOAs that measured 6–7 cm or more were managed surgically more often than those 5 cm or smaller, which were managed conservatively with antibiotics [26]. Older women were more likely to fail antibiotic treatment [27]. Moreover, elevated inflammatory markers such as white blood cell count > 16,000/µL, C-reactive protein, and erythrocyte sedimentation rate levels, in addition to higher body temperature, were shown to be major determinants for drainage or surgical treatment [28, 29].

This study focused on the effect of unilateral or bilateral cases of TOA to determine whether the course of treatment, could be predicted based on this identifying feature.

## Methods

This was a retrospective, cohort study. Data were retrieved from hospital electronic medical records from 2003 to 2017 of women admitted to the Gynecology Department of a tertiary level university-affiliated medical center.

### Ethics

This study was performed in accordance with the principles of the Declaration of Helsinki. All methods were carried out in accordance with relevant guidelines and regulations. All experimental protocols were approved by the Meir Medical Center Ethics Committee, approval number MMC-082-17. Due to the retrospective nature of the study, Meir Medical Center Ethics Committee has waived need for informed consent statement.

Patients diagnosed with either unilateral or bilateral TOA were included in the study. Criteria for diagnosis included a positive computerized tomography (CT) or ultrasound scan revealing adnexal infiltration and at least one of the following criteria: temperature > 38 °C, leukocytosis > 15,000 mm^3^, or surgically proven TOA. Excluded from the study were patients who did not meet the strict criteria or those with insufficient data in the electronic medical record.

Hemodynamically stable women were treated conservatively with broad-spectrum parenteral antibiotics according to the protocol followed by the hospital gynecology department and based on CDC guidelines [30]. All women admitted to the gynecology ward receive treatment comprised of intravenous amoxicillin/clavulanic acid 1 g, three times daily, along with oral doxycycline 100 mg twice daily and oral metronidazole 500 mg three times a day. Cervical swabs are used to collect material for polymerase chain reaction (PCR) tests for sexually transmitted diseases and bacterial infections. The continuation of doxycycline treatment is contingent on the identification of chlamydia infection in the PCR results. Antibiotic treatment is then modified based on the outcome of the swab results. Surgery was indicated for unstable women or in cases of failed antibiotic therapy after 72 h. Signs of continuous abdominal pain, elevated temperature, or persistently high levels of inflammatory markers while receiving parenteral antibiotic treatment were considered failure to respond to antibiotic treatment. Surgical interventions included subcutaneous drainage guided by ultrasound or CT, laparoscopic unilateral or bilateral salpingectomy, and in some cases, bilateral or unilateral salpingo-oophorectomy, with or without hysterectomy.

Demographic data, sonographic results, pre-operative, intraoperative, and post-operative findings were retrieved from the hospital electronic medical records to assess the differences between the two groups. No direct human involvement was needed.

Outcomes were compared between patients with unilateral TOA or bilateral TOA.

### Statistical analysis

Patient characteristics were compared between the groups, using student t-test for continuous variables, and the chi-square or Fisher’s Exact Test for categorical variables. Results were considered significant when the p-value was ≤ 0.05. Data are presented as number and percentage for categorical variables and as mean and standard deviation (SD) for continuous variables. All statistical analyses were performed using IBM SPSS for Windows, version 29.

A logistic regression was conducted, in which the type of treatment served as the dependent variable, while maternal age, parity, history of TOA, and bilateral or unilateral TOA, served as independent variables.

## Results

**Fig. 1:**
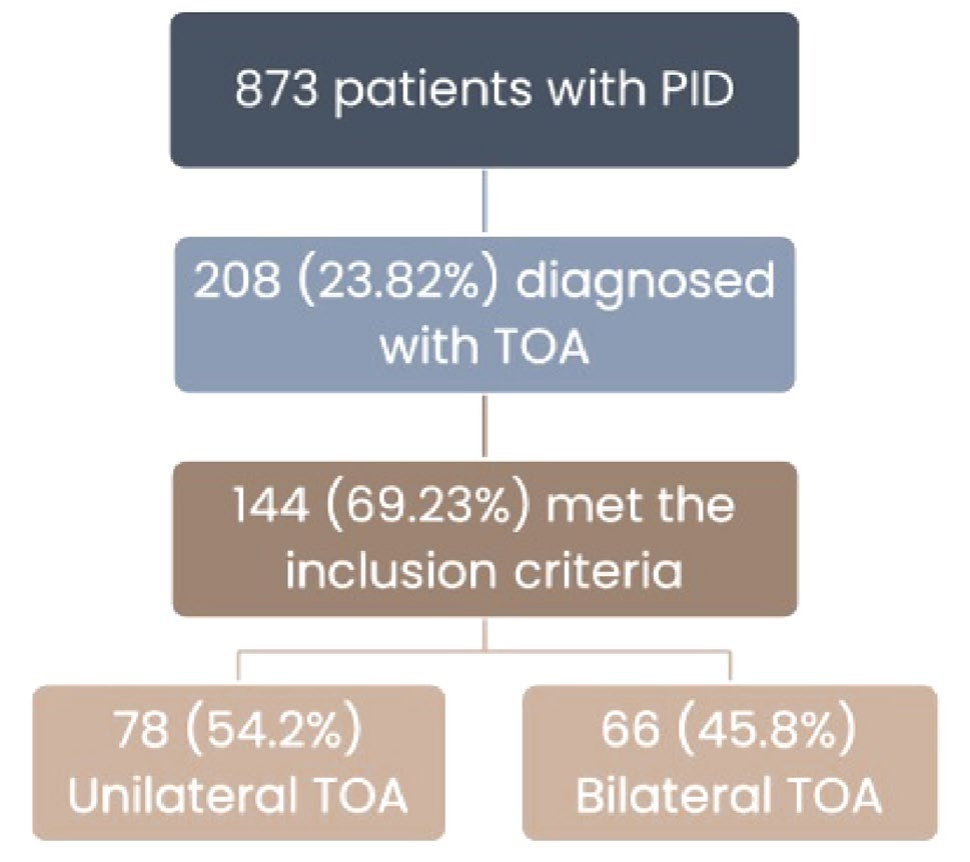
Study flow diagram. Flow diagram demonstrating the cohort included for analysis. We included 873 women for analysis who were admitted with PID, 208 of whom were diagnosed with TOA. Only 144 who met the strict criteria were included in the final cohort. Among them, 78 (54.2%) were diagnosed with unilateral TOA and 66 (45.8%) with bilateral TOA.

The research was based on data from 873 women diagnosed with PID. During this period, 208 (23.82%) were diagnosed with TOA. The final study cohort included 144 (69.23%) women who met the strict inclusion criteria, of whom, 78 (54.2%) were treated for unilateral TOA and 66 (45.8%) for bilateral TOA (Fig. 1).

Baseline demographics of age, marital status, gravity, parity, or comorbidities did not differ significantly between groups (Table 1).

**Table 1:** Women’s characteristics

Characteristic	Tubo-ovarian abscess status		*P-*value
	Unilateral (78)	Bilateral (66)	
Age, years; mean (± SD)	43.6 (± 10.8)	41.8 (± 10.1)	0.3
**Marital status, n (%)**			
Married	53 (69.7%)	44 (68.8%)	0.99
Other	23 (30.3%)	20 (31.2%)	
Gravidity, mean (± SD)	3.1 (± 2.3)	2.8 (± 1.9)	0.32
Parity, mean (± SD)	2.4 (± 1.7)	1.9 (± 1.3)	0.05
**Comorbidity, n (%)**			
None	61 (79;2%)	48 (77.9%)	
Cancer	4 (5.2%)	1 (1.6%)	
Sexually transmitted disease	2 (2.6%)	0 (0%)	0.24
Diabetes mellitus	3 (3.9%)	2 (3.2%)	
Endometriosis	7 (9.1%)	12 (19%)	

Pearson’s chi-square or t-test, as appropriate

Table 2 shows the prevalence of several risk factors in the groups, including history of abdominal surgery, smoking, contraception, history of PID, and number of PID episodes. Neither unilateral nor bilateral TOA had a significantly increased association with prior abdominal surgery, smoking, or contraception use. However, the results revealed a trend that those with bilateral TOA were more likely to have a history of PID than those with unilateral TOA (35.9% vs. 24.7%, respectively; p = 0.074).

**Table 2:** Risk factors for tubo-ovarian abscess

Risk factor	Tubo-ovarian abscess status		P*-*value
	Unilateral (78)	Bilateral (66)	
History of abdominal surgery, n (%)	36 (46.2%)	26.(40%)	0.46
Smoking, n (%)	21 (36.2%)	24 (49%)	0.18
Contraception, n (%)	32 (42.7%)	18 (30.5%)	0.3
History of PID, n (%)	19 (24.7%)	23 (35.9%)	0.07
No. of PID episodes, mean (± SD)	0.35 (± 0.74)	0.53 (± 0.85)	0.18

PID, pelvic inflammatory disease

Pearson’s chi-square or t-test, as appropriate

Table 3 shows that women who were diagnosed with bilateral TOA were more likely to undergo surgical treatment than those diagnosed with unilateral TOA (56.9% vs. 42.3%, respectively; p = 0.04). There was no difference in of the size of the TOA between groups.

**Table 3:** Disease characteristics in each group

Characteristic	Tubo-ovarian abscess status		P*-*value
	Unilateral (78)	Bilateral (66)	
**Diagnostic tool, n (%)**			
Ultrasound	36 (46.2%)	32 (50.2%)	
CT	37 (47.4%)	27 (42.2%)	0.8
Surgery	5 (6.4%)	5 (7.8%)	
Size of TOA, mm	6.5 (± 1.96)	7.05 (± 2.2)	0.2
**Treatment, n (%)**			
Antibiotics	45 (57.7%)	28 (43.1%)	0.04
Surgery	32 (42.3%)	37 (56.9%)	

TOA, tubo-ovarian abscess

Pearson’s chi-square or t-test, as appropriate

Multivariant logistic regression analysis found bilateral TOA was independently associated with surgical intervention(OR 2.5 (95% CI 1.17–5.29), p < 0.017).

## Discussion

Many efforts to characterize the predictive factors of whether to treat TOA with surgery as opposed to antibiotics alone, are underway. Markers of the TOA such as unilateral or bilateral involvement and size, as well as information about the patient, such as age and severity of inflammation, have traditionally been used as a guide for treatment. In this study, we evaluated the implications of one parameter in particular: unilateral or bilateral involvement, to observe its potential role in determining treatment and its correlation to certain risk factors.

We found a trend that showed a greater need for surgical treatment in women diagnosed with bilateral TOA in comparison to unilateral TOA, similar to the findings reported in previous research [20].

We did not observe a difference in the size of the abscess between those treated for unilateral compared to bilateral abscesses. This finding differs from several previous studies that reported a positive correlation between abscess size and need for surgical intervention [20, 25, 26, 31]. In fact, a study conducted by Levin et al., which attempted to build a novel risk assessment score to identify surgical indications for TOA, went as far as to suggest that abscess diameter was one of the strongest predictors of failed antibiotic treatment. Characteristics applied towards calculating the score included age, leukocyte count, abscess diameter, and presence of a bilateral abscess. Data revealed that age > 36 years at admission was associated with an odds ratio (OR) of 2.1, mean leukocytosis at admission > 16,000 mm^3^ with an OR of 2.2, and presence of a bilateral abscess with an OR 2.2. In comparison, ultrasonographic measurement of abscess diameter > 7 cm was associated with an OR of 3.6 [24]. In our study, however, unilateral or bilateral involvement was noted to be a better predictor of the type of intervention than the size of the TOA because size did not seem to differ between groups, whereas treatment methods did. It is important to note that the sample sizes in these studies were similar to those in the current study.

This information should be considered in clinical scenarios, as it could help physicians determine the most appropriate treatment for TOA and prevent unnecessary delays.

Despite the accepted practice of tailoring the surgical approach to the woman’s reproductive status, or unstable patients [32], there is a lack of understanding and consensus regarding the preferred procedure according to whether the abscess is unilateral or bilateral. The literature lacks clear guidance on the selection of a surgical approach, especially in the context of unilateral or bilateral TOA. While hysterectomy and bilateral salpingo-oophorectomy are recognized as definitive treatments, there is a knowledge gap regarding the extent of surgery needed for each case based on the surgical findings. There are various surgical interventions for treating TOA following failure of broad-spectrum antibiotics, as detailed in the Methods section. Drainage interventions can be guided by ultrasound or CT, while surgical procedures may involve unilateral or bilateral salpingectomy, unilateral or bilateral salpingo-oophorectomy, and in some cases, hysterectomy but there are no specific guidelines [33] Our data show that drainage was more successful for unilateral TOA, while surgical procedures of all kinds required more often for patients with bilateral TOA. It is important to note that this study did not delve into determining a preferred surgical treatment method. Instead, it highlighted a correlation between whether the disease was unilateral or bilateral and the choice of surgical intervention.

There are several strengths to the current study. First, to the best of our knowledge it is the first study to investigate the differences between bilateral and unilateral TOA. In addition, we used strict diagnostic criteria, only selecting cases with a white blood cell count above 15,000 mm^3^, a formal CT or US scan showing TOA, and a documented body temperature above 38 °C. Moreover, the study was conducted at a single medical institution. Therefore, decisions for each case were based on the same protocols, which did not change over the study period.

The study is not without limitations. Some are inherent to the retrospective study design. The data were based on medical records, which implies that some data could have been erroneously input by physicians or overlooked during review. Additionally, previously documented risk factors for TOA and blood samples could not be measured as those data were missing from the electronic medical records. Moreover, the relatively small sample size limited us from fully studying and understanding the effects of bilateral TOA.

## Conclusion

This retrospective study detected a trend toward more need for surgical treatment in women diagnosed with bilateral TOA and more successful outcomes with antibiotic treatment only among women with unilateral TOA.

It is plausible to consider that these findings may contribute to optimizing treatment decisions for TOA, potentially leading to a reduction in the duration of hospitalization, antibiotic exposure, and resistance. However, it is important to acknowledge that the results of the current study have limitations and further research is warranted to validate these potential outcomes.

## Data Availability

The datasets used and/or analyzed during the current study are available from the corresponding author on reasonable request.

## List of abbreviations

CT: Computed tomography
OR: Odds ratio
PID: Pelvic inflammatory disease
PCR: Polymerase Chain Reaction
TOA: Tubo-ovarian abscess
